# Pupil size and search performance in low and high perceptual load

**DOI:** 10.3758/s13415-018-00677-w

**Published:** 2018-12-14

**Authors:** Manuel Oliva

**Affiliations:** 1 Cognitive Science, Lund University, Lund, Sweden; 2 MAPP, Aarhus University, Aarhus, Denmark

**Keywords:** Perceptual load, Pupillometry, Locus coeruleus, Visual search, Attention

## Abstract

The ability to focus on a task while disregarding irrelevant information is an example of selective attention. The perceptual-load hypothesis argues that the occurrence of early or late selection mechanisms is determined by task-relevant perceptual load. Additionally, evidence shows that pupil size serves as proxy of locus coeruleus-norepinephrine (LC-NE) activity, a system associated with cognitive and attentional mediation. Here, we assessed pupil baseline (and pupil dilation) as predictors of load-related early and late selection performance. Participants were asked to search for a target in conditions of high and low perceptual load, while ignoring irrelevant stimuli. The results showed that pupil baseline size, measured prior trial onset, significantly predicted the upcoming search efficiency only in low perceptual load, when—according to the perceptual-load hypothesis—all perceptual information receives attentional resources. In addition, pupil dilation was linked to the time course of perceptual processing and predicted response times in both perceptual load conditions, an association that was enhanced in high load. Thus, this study relates attentional selection mechanisms, as defined by the perceptual-load theory, with pupil-related LC-NE activity. Because pupil baseline predicted attentional performance in low load but not in high load, this suggests that different attentional mechanisms are involved, one in which the LC-NE system plays a key role (low load) and one in which it is less relevant (high load). This suggests that the degree with which LC-NE influences behavioral performance is related to the perceptual load of the task at hand.

## Introduction

When a person is engaged in studying, playing sports, or focused on reading this article, that individual is likely to become simultaneously unaware of events happening in the surroundings. These examples of selective attention occur as the result of processing limitations, where either due to bottom-up or top-down mechanisms, only a limited amount of the information received from the environment is fully processed for meaning. An increasing body of evidence points to the importance of noradrenergic activity during perceptual processing. Therefore, this study investigates the link between visual perceptual load processing with locus coeruleus-norepinephrine function as measured through tonic and phasic changes in pupil size.

### Attention and pupil size

Fluctuations in pupil size have been associated with the time course of perceptual processing (de Gee et al., 2014) and decision-making (Einhäuser et al., 2010; Oliva & Anikin, 2018). This relationship arises because under isoluminance conditions, pupil dilation is largely caused by norepinephrine (NE) release from the locus coeruleus (LC) (Joshi et al., 2016). The LC sends inputs to different prefrontal brain areas involved in control functions and attentional processing (Foote et al., 1991; Joshi et al., 2016). Norepinephrine release on these target areas is believed to act by increasing neural gain (Aston-Jones & Cohen, 2005), which enhances the signal-to-noise ratio in the processing of sensory input (Sara & Bouret, 2012b; Mather et al., 2016a; Arnsten & Rubia, 2012). In fact, a recent neuroimaging study showed that LC-NE activity improves the precision of cortical representations of perceptual signals (Warren et al., 2016). Among other functions, the LC-NE system seems to be highly involved in the detection of behaviorally relevant stimuli. When a target is detected, the LC produces a phasic activation that is subsequently accompanied by a task-evoked pupil response (Usher, 1999; Aston-Jones et al., 1994; Privitera et al., 2010). The LC-NE system can have periods of higher or lower tonic (basal) activity, which have been associated with shifts in attentional performance (Usher, 1999; Gilzenrat et al., 2010). Most of this evidence comes from electrophysiological studies in monkeys performing go/no-go tasks, in which epochs of low LC tonic activity correlated with better attentional performance (reflected by lower rates of false-positive responses to non-target stimuli) and pronounced phasic spike bursts after the perceptual detection of the targets. On the contrary, high tonic activity correlated with poorer performance (higher rate of false-positive responses to non-target stimuli) and diminished LC-NE phasic responses.

Although accumulating evidence links noradrenergic activity with attention and cognitive processes, the role of LC-NE activity within attentional selection mechanisms is not yet understood. In the present study, we used pupil size measures of tonic and phasic LC-NE activity to predict performance in an attentional task.

### Perceptual load and the locus of selection

Researchers have long been interested in detecting how behaviorally relevant information is selected within the course of attentional processing. The first influential theory that accounted for selective attention was proposed by Broadbent (1958) and later updated by Treisman (1969). In this theory, they proposed a two- stage perceptual mechanism where first, physical features of the stimuli are extracted in parallel and filtered, so that only the stimulus of interest will receive further processing. According to this theory, selection occurs in an early processing stage after which irrelevant stimuli receive no further analysis.

Early selection models are well suited to explain selection in perceptually difficult tasks, such as in “shadowing” experiments (Cherry, 1953), which involve high perceptual load (i.e., complex target stimulus, large set size). In these experiments, participants had to hear two auditory messages played each on different ears (usually by means of headphones), and repeat out loud (or to “shadow”) only one of the messages. Along with early selection models, these experiments showed that individuals are good at efficiently selecting one channel while at the same time disregarding the irrelevant one (Treisman, 1969). However, this model failed to explain selection under low perceptual load (i.e., simple target stimulus, small set size). A clear example of the latter are flanker tasks (Eriksen and Eriksen, 1974) where participants are asked to report the presence of one out of two possible targets while at the same time ignoring a peripheral distractor. This paradigm shows that under low load, individuals are unable to ignore irrelevant stimuli, which translates into slower responses compared to when no distractor is present.

Deutsch and Deutsch (1963) proposed a model capable of explaining selection in tasks with low perceptual load, such as the flanker task. In contrast to early selection, this model posits that perception proceeds in parallel across all stimuli. According to this account, selection of the target stimulus occurs “late” in processing, as a result of the need to provide a pertinent behavioral response. Late selection models explain flanker interference effects by predicting that because of the absence of early perceptual filtering, irrelevant stimuli would compete with the target stimulus and influence response times.

These seemingly contradictory differences between models led (Kahneman & Treisman, 1984) to suggest the existence of two different attentional mechanisms acting in different circumstances, a hypothesis that was further developed in the perceptual load hypothesis (Lavie, 1995; Lavie & Tsal, 1994). The perceptual load model integrates early and late selection accounts by proposing that the perceptual load of the task at hand is the main factor determining whether early or late mechanisms will occur. As in the late account, it proposes that perception is an automatic process, in the sense that it proceeds in parallel across all stimuli without voluntary control. The perceptual load hypothesis adds that perception proceeds automatically only until the perceptual system runs out of capacity, in which case not all perceptual information receives further processing. By manipulating the degree of perceptual load of a flanker task, Lavie and Cox (1997) showed that high perceptual load can prevent the interference produced by a competing flanker. In addition, (De Fockert et al., 2001) showed that cortical functions are important for selective attention in conditions of low perceptual load. In such cases, all information is fully perceived and working memory seems to play a key role in maintaining the prioritization of relevant information. In a series of experiments, it was shown that by taxing the participants’ working memory system, selective attention was impaired in low load but not in high load (De Fockert, 2013; Lavie et al., 2004).

### The present study

**Fig. 1:**
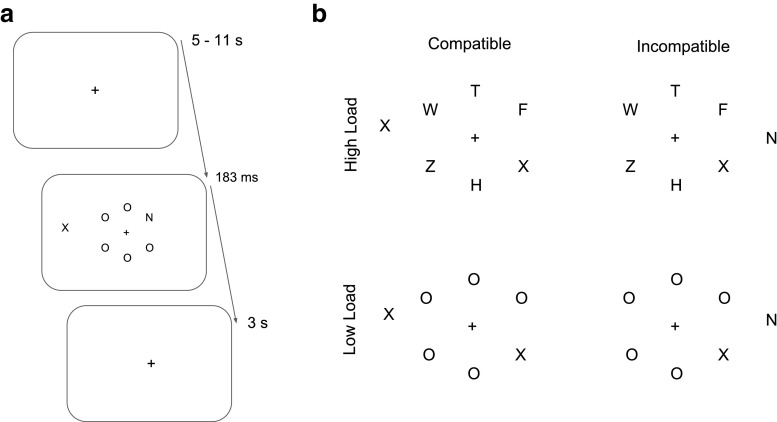
**a** Participants started fixating at a central cross and after a non-aging foreperiod a search array was presented and participants had to report the target stimulus. **b** Examples of the search arrays from each condition

In the present study, we examined the relationship between LC-NE activity—as measured through pupil size—and the efficiency of visual search for a target in conditions of high and low perceptual load. For such a purpose, we adapted a task previously used for the study of perceptual load and selective attention (Lavie, 1995; Lavie & Cox, 1997; Theeuwes et al., 2004) so that it could be used under isoluminant conditions. In this task, participants are instructed to report the appearance of a target letter (X or N) within a central search array that contains the target together with other five non-target letters (Fig. 1). Simultaneously, participants have to ignore a peripheral distractor. The distractor letter can be compatible (i.e., same as target letter) or incompatible (i.e., alternative target letter). The perceptual load of the task is manipulated by varying the similarity between the target with the non-target letters (Fig. 1). In high load, the non-target letters in the array are more similar to the target than in conditions of low load. In this way, perceptual load is manipulated while keeping similar set sizes between the low and high load search conditions.

As described above, this paradigm predicts that under low load, all stimuli from the search array will receive perceptual resources and analyzed in parallel. This leads to a condition where all perceptual information access awareness and selection is then resolved after stimuli identification—in which case, cognitive control and working memory are critical for successfully selecting and prioritizing relevant information (Lavie et al., 2004; De Fockert, 2013). In this context, we expect that visual search performance should be modulated by the LC-NE tonic activity reflected by baseline pupil size—measured just before trial onset—particularly in conditions of low perceptual load. If LC-NE modulates cognitive processing, this modulation should be reflected in low load, when all stimuli receive full perception. In high perceptual load, in contrast, not all perceptual information is perceptually processed at once. This is because perceptual information is filtered out due to capacity limits. In such a case, we expect pupil baseline size not to predict search performance.

The perceptual load of the main task will also influence distractor processing. Low perceptual load arrays may allow the perception of the distractor, which may interfere with response selection in the case of incompatible trials. High perceptual load displays, in contrast, will deplete resources and distractor compatibility should have little influence on response times. As such, the degree of distractor processing is an indirect measure of perceptual load effect, which reflects different attentional selection strategies (Lavie, 2010).

## Methods

### Participants

Nineteen participants (mean age = 26, age range = 21–41) with normal or corrected-to-normal vision voluntarily participated in the experiment and received a cinema ticket in return. Data from two participants were discarded due to poor eye-tracking data quality (more than 50% of data loss, see Data Analysis).

### Ethical statement

In accordance with the Swedish law (SFS 2003: 460, 16 §) all participants gave written consent for participating in the experiment. The present study was exempt from the requirement for ethical approval.

### Apparatus

The presentation of the stimuli was controlled using Psychopy (Peirce, 2007) (v2.85). The stimuli were presented on a 1280 × 1080 monitor screen (Samsung 931C) with a refresh rate of 75 Hz. Pupil size and gaze position were recorded with a tower-mounted eyetracker (SMI, Teltow, Germany) at 500 Hz. Participants used a chinrest and maintained a viewing distance of 65 cm. Isoluminant colors for the letters and the background were approximated using the YUV color encoding system and later adjusted to be perceptually isoluminant with the flicker-fusion procedure (Lambert et al., 2003). The resulting colors had the RGB values of 69, 149, 24 for the background and 223, 61, 61, for the letters. Under these conditions, the luminance was kept constant throughout the experiment at 56 *cd*/*m*2.

### Stimuli

The target letters that participants were instructed to report were X and N. In the low load condition, the non-target letters were all “O”. In the high load condition, the non-target letters were the letters “W”, “Z”, “F”, “H”, “T”. In this way, there were always five non-target letters in both the high and low conditions, although, the processing demands were higher for the high perceptual load condition (see Fig. 1). Each letter subtended 1.1° in height and 0.8° in width. The letters were presented randomly at 45°, 90°, 135° of arc on an imaginary hexagon at an eccentricity of 3.5°. The distractor letter was presented randomly to the left or right sides of the letters array with a random position varying between +/- 10° of arc. The distractor was displayed at an eccentricity of 4.5° from the fixation point.

### Design and procedure

Participants received 192 experimental trials separated in four blocks of 48 trials each with optional breaks in between blocks. Blocks of high and low load were presented in counterbalanced order. There was an equal number of compatible and incompatible trials on each block and the position of the distractor was randomized in every trial. Participants completed at least 48 practice trials. If necessary, the practice session was extended until participants reached at least 70% of correct trials on each load condition. After calibration of the eyetracker, the resting state baseline size was measured. For this purpose, participants were asked to passively fixate for 40 s on a central fixation circle. This prolonged window allowed to average out local fluctuations in pupil size, so as to calculate the mean pupil size for each participant when they are not engage in any specific cognitive task. The experimental trials began with the presentation of a fixation cross at the center of the screen, which was presented following a non-ageing foreperiod of 5-11 s. A non-ageing foreperiod reduces the effect of target onset expectations (Oswal et al., 2007). The relatively long foreperiod allowed the pupil to subside back to baseline levels. Subsequently, the central search array and distractor were displayed for 183 ms (see Fig. 1). The short presentation time was intended to avoid the use of eye movements for the visual search. If an X was presented, the participants had to press the “2” key; if an N was presented, they had to press the “0” key. Participants were instructed to report the target present in the central search array and to ignore the peripheral distractor. Feedback about their performance (response times and error rates) was displayed on the computer screen after the completion of each block.

#### Data analyses

The baseline pupil diameter for each trial was calculated as the average diameter over a period of 1 s before trial onset (during the inter-trial foreperiod). For the analysis of baseline, trial baseline values from each participant were normalized by the respective average resting state pupil size measured during 40 s of passive fixation (see Procedure), so as to compare the behavioral state of the participant prior trial onset against their resting state. Task-evoked pupil responses were computed as the relative difference in pupil size between the trial baseline and the peak pupil dilation measured until 3 s after the participant’s key press. Pupil dilation peak timing was determined as the difference in time between the peak pupil dilation and trial onset. Pupil data were processed in Python (2.7.11) to detect blinks and gaze displacement. Blinks and other artifacts where removed from the resting state baseline average calculation. Trials containing blinks between trial onset and the peak dilation and/or when gaze was displaced from the central fixation cross were excluded from all analyses. Trials in which periods of blinks, missing data and gaze displacement represented more than 20% of the total trial samples were also excluded. Under these criteria, two participants were excluded from the analyses for excessive data loss (less than 50% usable trials). All the included participants had above 74% of usable data. We used R (RStudio, v1.0.153) to perform the analyses of the relationship between response time and pupil size. Response times were positively skewed. A common approach to correct for deviations of normality is to inverse transform response times (1/RT), however, applying nonlinear transformations can affect the interpretation of interactions. Thus, we used generalized linear mixed-effect Bayesian models assuming an inverse Gaussian distribution with inverse (-1/RT) link, which provide a solution to this problem by satisfying normality assumptions without the need for transformation (Lo & Andrews, 2015). Because pupil dilation and pupil baseline were partially correlated (*r* = .39), their effects were assessed in two separate models. Errors rates were analyzed through logistic regressions. Statistical significance of predictors was tested with likelihood ratio tests using lme4 (Bates et al., 2015). To extract confidence intervals, we fit analogous Bayesian models, which arguably offer more robust estimates in the context of multilevel regression. Bayesian models were created in Stan (http://mc-stan.org/) and brms package (Bürkner, 2017). To improve convergence and guard against overfitting (McElreath & Smaldino, 2015), we specified mildly informative conservative priors. Python and R scripts for analyses are available in supplementary materials (osf.io/4r7wf).

## Results

### Perceptual load and classic interference

The attentional task utilized in this study was adapted from a commonly used visual search task for the study of perceptual load effects (Lavie 1995, 2005). In this task, stimuli are usually displayed without controlling for luminance. However, because pupil size reflects both LC-NE activity and the light reflex, we adapted this task so that the stimuli were isoluminant with the background. Isoluminance may reduce contrast between the stimuli and the background and therefore we first assessed whether the classic effects of perceptual load were maintained under our manipulation.

We hypothesized that perceptual load should modulate interference caused by a peripheral distractor (Lavie & Cox, 1997). In this, we expected an interaction between perceptual load and compatibility of distractor, where incompatible distractors should delay response times when they are processed (i.e., under low load where individuals still have available perceptual resources). In contrast, little or no distractor interference is expected in high perceptual load, where there is little spare capacity to process the peripheral distractor.

In order to analyze the independent effects of load and compatibility on response time, we conducted a linear regression analysis of response time. The model included load (high/low) and compatibility (compatible/incompatible) as main effects, random intercepts for participants, and random slopes for load.

**Table 1:** Response times (SE in parenthesis) for the different load and compatibility conditions

	Compatible	Incompatible	I - C
Low Load	701 (35)	729 (34)	27^∗^
Errors (%)	1.5	2.9	−
High Load	1008 (40)	1003 (46)	-5
Errors (%)	14.3	14.8	−

There was a significant interaction between load and distractor compatibility, indicating that our manipulation successfully reproduced the effect of perceptual load on irrelevant distractor processing. Error rates (in percentage) are presented for each condition. * *p*< 0.01

The results showed that, as expected, low-load displays yielded significantly faster RTs than did the high load (1005 vs. 715 ms; *χ*^2^ = 22.52, *df* = 1, *p*<0.001). The effect of compatibility of the distractor had no significant main effect (853 vs. 840 ms; *χ*^2^ = 2.28, *df* = 1, *p* = 0.131). However, in line with the perceptual load theory, there was a significant interaction between load (high vs. low) and type of distractor (compatible vs. incompatible) (Table 1). In high load the compatibility effect was only - 5 ms, while in low load it was 27 ms, indicating an interaction estimate of 33 ms (*χ*^2^ = 7.45, *df* = 1, *p* = 0.006). Total error rates were relatively low (9 %). There were less errors in the low load than in the high condition (*χ*^2^ = 28.20, *df* = 1, *p*<0.001). Compatibility did not affect error rates (*χ*^2^ = 1.38, *df* = 1, *p* = 0.236) and there was no interaction between load and compatibility (*χ*^2^ = 3.19, *df* = 1, *p* = 0.068). Overall, these results indicate that we were successful in designing isoluminant high- and low-load conditions that resulted in compatibility effects in the low-load condition and a no compatibility effect in the high-load condition.

### Pupil baseline vs. perceptual load

**Fig. 2:**
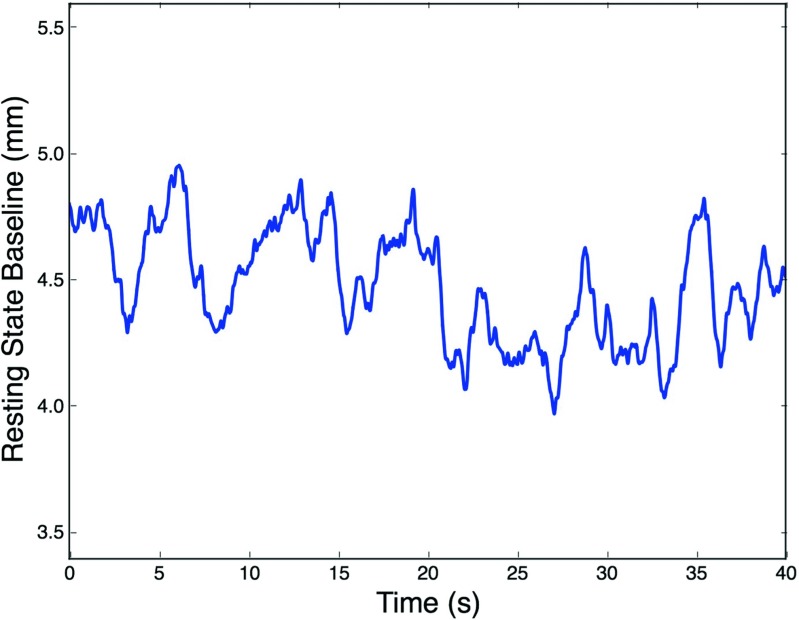
Pupil size fluctuations during passive fixation and isoluminance conditions. Spontaneous changes in pupil size correlate with to LC-NE activity (Joshi et al., 2016). An average resting state baseline was extracted from each participant in order to normalize pupil size

Pupil baseline fluctuations (see Figures 2 and 6) have been shown to be an indicator of LC-NE tonic activity (Joshi et al., 2016). LC-NE can have periods of higher or lower basal activity, which have been associated with shifts in attentional performance (Aston-Jones, 2005). In particular, low tonic LC activity was linked with better performance in attentional tasks (Aston-Jones & Cohen, 2005; Gilzenrat et al., 2010). Therefore, if the pupil baseline preceding each trial reflects activity in the LC, we hypothesized that it should have a significant effect on search efficiency.

**Fig. 3:**
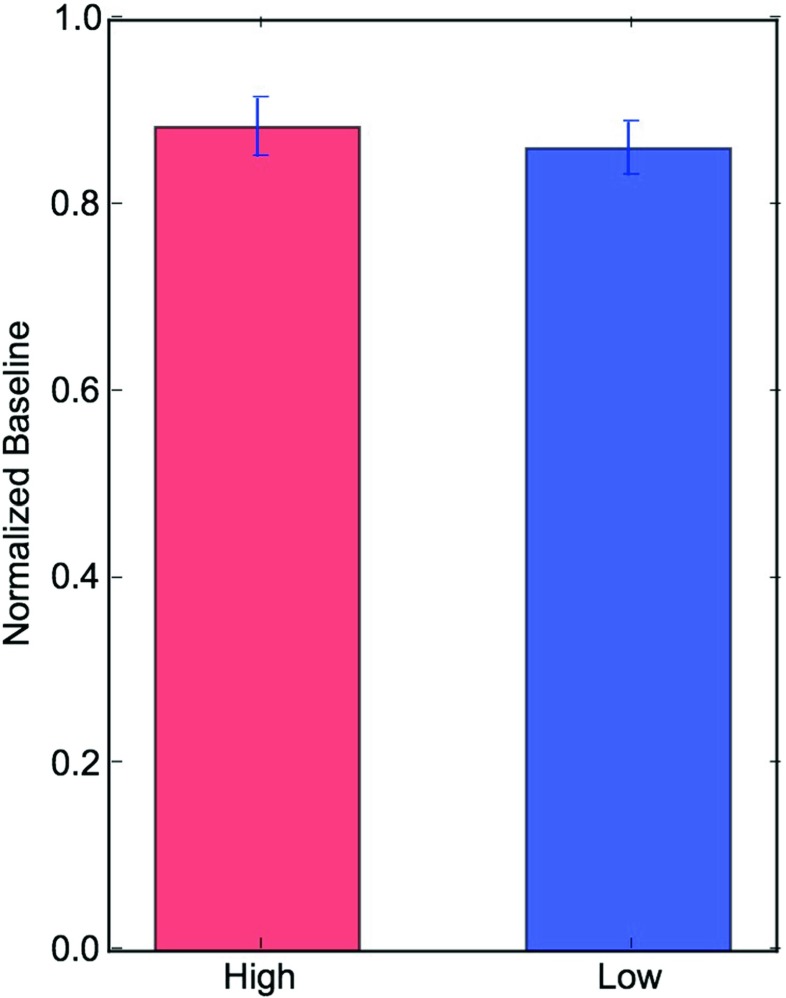
The pupil baseline prior the onset of each trial was normalized by a resting state baseline recorded during passive fixation. Trial baselines preceding each trial were on average smaller compared to a resting state baseline. However, there were no differences between conditions. Such decrease in pupil size seems to reveal attentional predispositions towards the upcoming attentional task. *Error bars* represent SEM

In order to compare the effect of baseline, the baselines preceding each trial were normalized by a resting state pupil size recorded after the calibration procedure of the eyetracker (see Fig. 2). As shown in Fig. 3, the baseline preceding trial onsets were smaller in both the low (0.885, 95% CI [0.945; 0.824]) and high (0.862, 95% CI [0.916; 0.807]) perceptual load conditions compared to a resting state baseline. However, a comparison between load conditions showed that their baselines did not differ significantly between each other (*χ*^2^ = 0.703, *df* = 1, *p* = 0.402). A transition towards a smaller pupil size suggests a decrease in LC tonic activity, usually associated with improved attentional performance. In the case of our study, this shift suggest some attentional predisposition of the participants towards the upcoming attentional task.

### Baseline as predictor of response time

We then investigated whether fluctuations in pupil baseline would predict search efficiency (as reflected by response times). To tease apart the independent effect of baseline, we conducted a linear regression analysis that included normalized trial baseline, perceptual load, and distractor compatibility as fixed effects. The model also controlled for trial order effects and included random intercepts for participants and random slopes for load.

The results indicated that the effect of the interaction between perceptual load and distractor compatibility, now controlling for pupil baseline, was found to remain significant compared to when no pupil size information was included (*χ*^2^ = 9.29, *df* = 1, *p* = 0.002). In fact, a comparison between models showed that adding pupil baseline information significantly improved the model’s fit (*χ*^2^ = 18.91, *df* = 2, *p*<0.001).

**Fig. 4:**
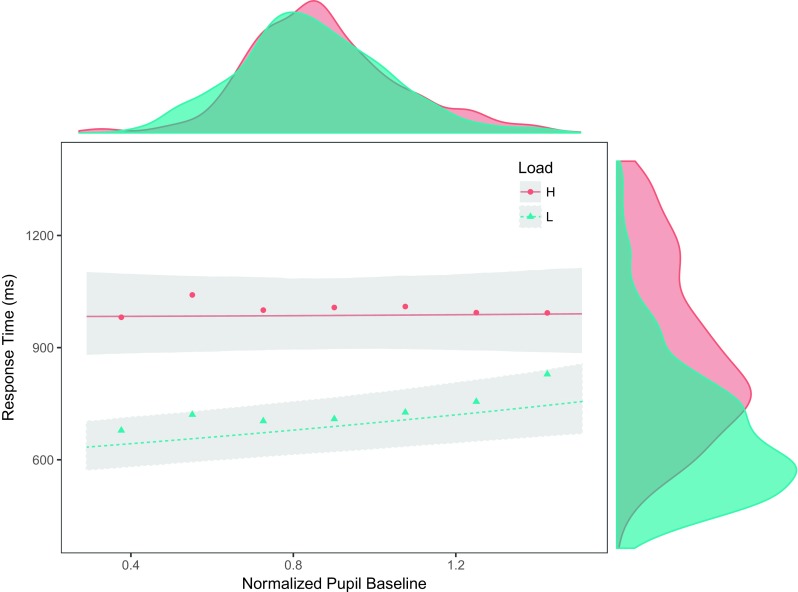
The effect of baseline pupil size on response time for high (H) and low (L) perceptual load. There was a significant relationship between pupil baseline and response time only in low perceptual load. This difference suggest the involvement of distinct attentional mechanisms, where pupil baseline seems to only reflect the influence one of such mechanisms. The plot displays fitted values and 95% credible intervals obtained from a Bayesian mixed model. Each data point represents aggregated reaction time data. For display purposes together with model data, RTs were centered around each participant’s average response time and around the respective perceptual load group average. Smoothed RT histograms were restricted to fit plot limits

As shown in Fig. 4, there was a significant interaction between load and pupil baseline in explaining search performance (*χ*^2^ = 9.29, *df* = 1, *p* = 0.002). This interaction revealed an enhanced effect of baseline in conditions of low perceptual load. In low load, the effect of baseline going from a minimum to a maximum size is to slow down RTs by 121 ms (95% CI [62; 190] ms), where the smaller the baseline, the faster the participant’s RTs. In contrast, the baseline did not predict search efficiency in high load (*χ*^2^ = 0.164, *df* = 1, *p* = 0.685). The interaction between baseline and compatibility was not reliable (*χ*^2^ = 2.31, *df* = 1, *p* = 0.128).

In addition to slowing down responses, pupil baseline did not significantly increase the log odds of committing an error (*χ*^2^ = 2.17, *df* = 1, *p* = 0.141). This indicates that the shift in response times cannot be attributed to a speed/accuracy trade-off.

### Task-evoked pupil dilation

**Fig. 5:**
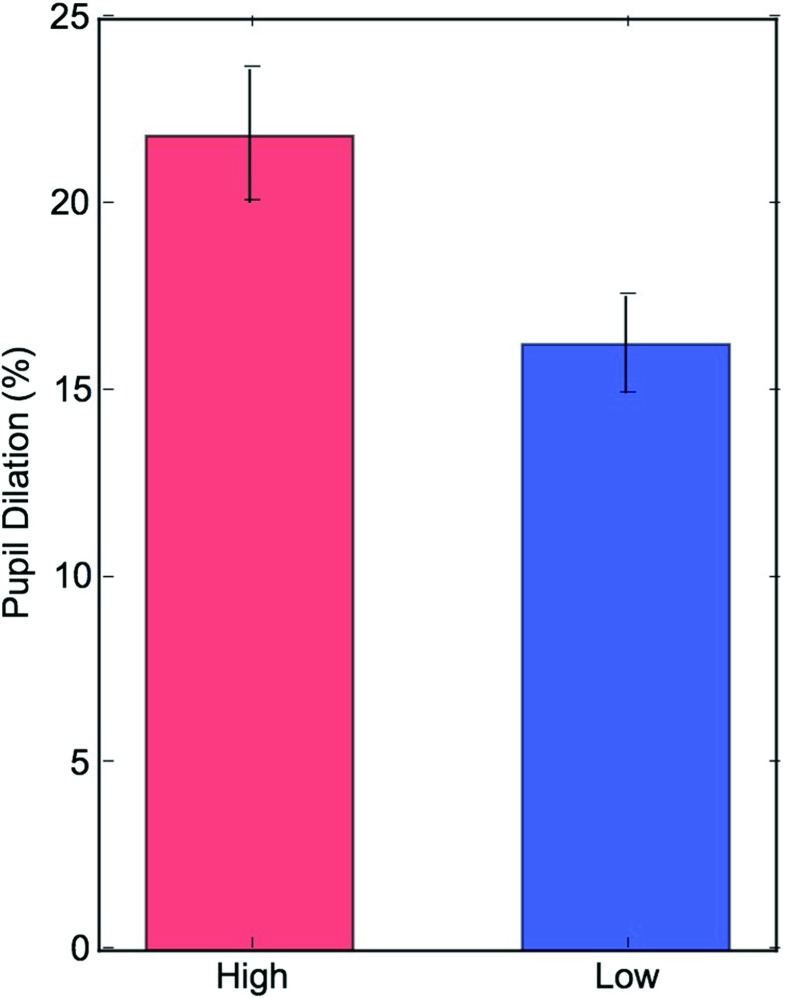
The figure shows pupil dilation relative to baseline for low and high perceptual load. The amplitude of pupil dilation was larger in high load (*p*<0.001). *Error bars* denote SEM

**Fig. 6:**
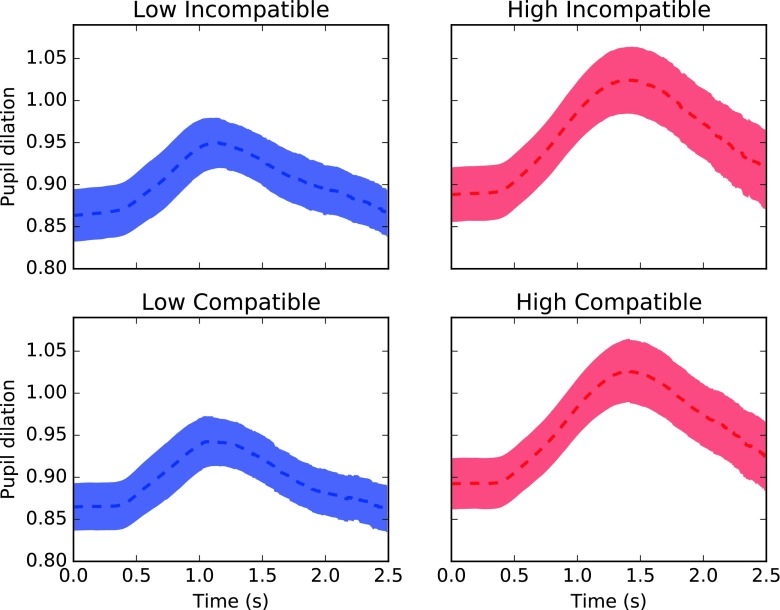
Pupil responses for each condition. For visualization purposes, pupil responses are shown relative to resting baseline size. Pupil responses are time-locked to the moment of stimulus onset (0 s). As shown in the figure, pupil baselines prior trial onset were on average smaller than during passive fixation. *Shaded areas* represent SEM at each time point

Task-evoked pupil responses were analyzed with a regression model that shared the same structure as that for pupil baseline. As depicted in Figs. 5 and 6, high perceptual load caused significantly larger peak pupil dilations than low load (16 vs. 21%, *χ*^2^ = 102.58, *df* = 1 *p*<0.001).

**Fig. 7:**
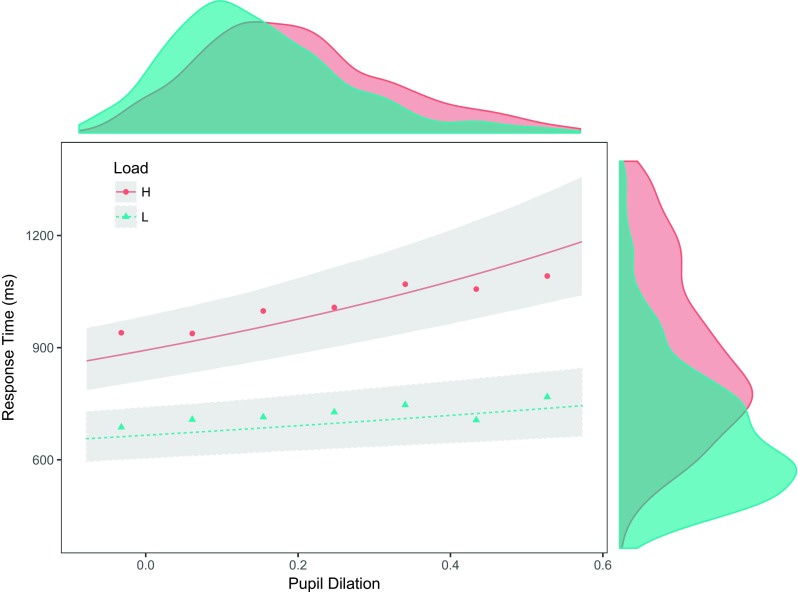
Pupil dilation and response times for high (H) and low (L) load. There was a positive relationship between pupil dilation and response times in both conditions, a relationship that was enhanced in conditions of high perceptual load. The plot displays fitted values and 95% credible intervals obtained from a Bayesian mixed model. Each data point represent aggregated reaction time data. RTs were centered around each participant’s average response time and around the respective perceptual load group average. Because of uneven pupil dilation tails, 1.5% of data was excluded only for aggregation and display purposes. Smoothed RT histograms were restricted to fit plot limits

The analyses showed that there was a significant effect of pupil dilation on response time, where larger pupil dilations correlated with slower response times (*χ*^2^ = 78.61, *df* = 1, *p*<0.001), which can be observed in Fig. 7. As for the baseline, there was a significant interaction between pupil dilation and perceptual load, where the role of pupil dilation was enhanced in predicting response times in high perceptual load (*χ*^2^ = 9.39, *df* = 1, *p* = 0.002). The effect of pupil dilation going from a minimum to a maximum is to slow down RTs by 110 ms (95% CI [47; 187]) in low load, and 558 ms (95% CI [377; 807] in high load. This indicates that the amplitude of pupil dilation was positively related to the latency of the response, where slower RTs led to larger pupil dilation. Lastly, there were no differences in the amplitude of pupil dilation as function of distractor compatibility (*χ*^2^ = 1.069, *df* = 1, *p* = 0.301).

Larger pupil dilations were associated with an increase of 3.97 in the log odds of making an error (*χ*^2^ = 18.35, *df* = 1, *p*<0.001).

### Pupil peak dilation timing

The timing to peak dilation followed a dynamic similar to that observed for pupil dilation amplitudes reported in the previous section. There was a main effect of peak dilation time in predicting response times (*χ*^2^ = 188.54, *df* = 1, *p*<0.001), indicating that the timing of pupil dilation followed the timing of decoding and responding to the target letter. There was also a significant interaction with load (*χ*^2^ = 72.26, *df* = 1, *p*<0.001), revealing that the correlation between peak pupil dilation timing and the behavioral response was enhanced in high load.

## Discussion

This study is the first to relate LC-NE function, as measured through pupil size, with search performance in conditions of high and low perceptual load. According to recent evidence, fluctuations in pupil size serve as a proxy of LC-NE activity (Joshi et al., 2016), which, in turn, is associated with cognitive and attentional mediation (Aston-Jones & Cohen, 2005). The analyses presented here show that the extent at which pupil size fluctuations predicted task performance was modulated by task load. Specifically, pupil baseline predicted response times only in low load, whereas task-evoked pupil dilation predicted response times both in low and high load, although this relationship was enhanced in high load. In addition, the timing of the task-evoked pupil response also predicted search performance in both conditions.

LC activity is believed to modulate arousal and cortical functions, with extensive influence over behavior and cognitive states (Aston-Jones & Cohen, 2005). Indeed, recent reviews suggest an important role of transient fluctuations in arousal on the modulation of cognitive and learning processes (Sara & Bouret, 2012a; Eldar et al., 2013; Mather et al., 2016b). The present analysis of pupil baseline shows that there was a significant decrease in pupil size as participants engaged in the experimental tasks (Fig. 3) compared to when pupil size was measured during passive fixation (Fig. 2). This finding is in line with previous evidence showing that low tonic LC-NE activity (also revealed by relatively smaller pupil size) correlates with periods of good attentional performance in go/no-go tasks (Gilzenrat et al., 2010; Usher, 1999) as well as with improved cortical representations of sensory inputs (Warren et al., 2016). Therefore, we interpret these results as suggesting that shifts in pupil baseline size reveal attentional preparatory mechanisms in anticipation for perceptual processing.

The LC presents spontaneous fluctuations in tonic level that translate into changes in pupil size, as observed during passive fixation (see Figs. 2 and 6). Here, we show that this variability in baseline size across trials predicted search performance, specifically in conditions of low perceptual load. In the context of a visual search task, low perceptual load allows participants to perform an efficient search for the target (Lavie & Cox, 1997), where all stimuli receive perceptual resources and information can be extracted in parallel across stimuli. Contrary to conditions of high perceptual load, selection in low perceptual load has been shown to rely more on control functions. For instance, decreases in attentional performance were reported, specifically in low perceptual load, when working memory capacity is taxed (De Fockert, 2013; Lavie, 2010). This interaction between control functions and the degree of perceptual load may explain the results presented here between pupil baseline and perceptual load. If selection in low load is more dependent on cognitive control, and cortical functions are modulated by the LC-NE system, then a modulation of performance in low load is to be expected.

Recently, the perceptual load model was rivaled by an alternative account suggesting that the reduction in distractor interference under high perceptual load is due to ‘dilution’ of the distractor within the irrelevant letters in the search array (Tsal & Benoni, 2010; Benoni & Tsal, 2013; Wilson et al., 2011; Cave & Chen, 2016). Although both models differ on the selection mechanisms involved in high load, both agree on the fact that distractors are more likely to be processed under low perceptual conditions, as all information is perceived at once. Regardless of the differences between these views, the results presented here fit with predictions derived from both the perceptual load and dilution accounts, in the sense that they denote the occurrence of different attentional effects.

Pupil baseline predicted search efficiency—as reflected by faster response times—and this was not connected to changes in response error rates. First, this is indicative that the improvement in response times cannot be attributed to speed/accuracy trade-off mechanisms. Secondly, this contrasts with experiments in monkeys using a go/no-go task which report that high tonic LC activity were associated with increased false positives responses (Aston-Jones et al., 1994; Usher, 1999; Gilzenrat et al., 2010). This difference may rely in the fact that “go” responses in such tasks had to be performed within strict time constraints, forcing speeded responses. In addition, the task used in the present study differs from go/no-go paradigms in the sense that it assesses attention allocated across stimuli within a search display, rather than the monitoring of a rapid visual stimuli presentation.

### Pupil dilation amplitude and timing

The processing of the search arrays was followed by a phasic increase in pupil size. In line with previous reports (Lisi et al., 2015; Porter et al., 2006; Wahn et al., 2016), higher perceptual load elicited larger pupil dilation amplitudes (Fig. 7). In addition, the present results revealed an interaction between load and pupil responses in predicting search performance. While the timing and amplitude of pupil dilation were significant predictors of response time in both low and high, the link between pupil dilation and response time was significantly more pronounced in conditions of high load, when the system was perceptually overloaded (see Fig. 7).

Pupil dilation has generally received more attention than pupil baseline in psychological research. For instance, pupil dilation has been long associated with memory load and mental effort (Kahneman & Beatty, 1966). In particular, several articles reported that pupil dilation reflects the time course of decision-making during perceptually challenging tasks involving both visual (de Gee et al., 2014; Einhäuser et al., 2008) and affective processing (Oliva & Anikin, 2018). In line with these previous reports, the results presented here show that both the time course and amplitude of pupil dilation predicted the timing of the participants’ responses. In addition, we show that this link between pupil dilation and response time was enhanced in high load. However, task-evoked responses present some limitations compared to pupil baseline measures. Whereas task-evoked pupil dilation is measured during task processing and even after participants have responded (because of its slow latency), pupil baseline is measured right before task onset, therefore providing actual anticipatory information.

Pupil dilation has been shown to provide short latency information about target processing of up to 100 ms, despite the pupil’s slow latency dynamics (Zylberberg et al., 2012). However, the temporal resolution of pupil responses did not allow us to find significant differences in the timing and amplitude between compatible and incompatible trials, with temporal differences between conditions largely below such value. Incompatible trials where the distractor produces response interference were expected to elicit larger pupil responses as this interference may increase cognitive and attentional demands to elucidate the actual target. Although there was an interaction between load and response time reflecting interference effects, no differences where found in terms of pupil dilation amplitude and peak timing.

All in all, the results of the present study reveal an interaction between pupil baseline and attentional performance as a function of perceptual load that is in line with the perceptual-load hypothesis and with current views of LC-NE function (Aston-Jones & Cohen, 2005). The results show that the degree with which LC-NE influences behavioral performance is related to the perceptual load of the task at hand. Thus, this study links early and late selection mechanisms, as defined by the perceptual-load hypothesis, with LC-NE function, as measured by pupil size.

## Conclusions

In this study, we focused on pupil baseline measures as predictors of visual selection performance in conditions of high and low visual perceptual load. The results indicate that pupil baseline only predicts selection performance in conditions of low perceptual load, where all perceptual information presented in the display can be processed in parallel. The fact that baseline predicts visual search performance only in low load, reflects the involvement of different attentional processes, one that seems to be mediated by the LC-NE system and one that is not. In line with previous studies, the time course of pupil responses did also reflect the timing of perceptual processing in both high and low perceptual load, but this relationship was enhanced in high load.
